# Reliability and Validity of an Inertial Measurement System to Quantify Lower Extremity Joint Angle in Functional Movements

**DOI:** 10.3390/s22030863

**Published:** 2022-01-23

**Authors:** Zhenyu Shuai, Anqi Dong, Haoyang Liu, Yixiong Cui

**Affiliations:** 1 Sports Coaching College, Beijing Sport University, 48 Xinxi Road, Beijing 100084, China; shuaizhenyu@126.com; 2 AI Sports Engineering Lab, School of Sports Engineering, Beijing Sport University, 48 Xinxi Road, Beijing 100084, China; daq5213@126.com (A.D.); cuiyixiong@bsu.edu.cn (Y.C.)

**Keywords:** inertial sensors, kinematics, functional activity, validity, repeatability

## Abstract

The purpose of this research was to determine if the commercially available Perception Neuron motion capture system was valid and reliable in clinically relevant lower limb functional tasks. Twenty healthy participants performed two sessions on different days: gait, squat, single-leg squat, side lunge, forward lunge, and counter-movement jump. Seven IMUs and an OptiTrack system were used to record the three-dimensional joint kinematics of the lower extremity. To evaluate the performance, the multiple correlation coefficient (CMC) and the root mean square error (RMSE) of the waveforms as well as the difference and intraclass correlation coefficient (ICC) of discrete parameters were calculated. In all tasks, the CMC revealed fair to excellent waveform similarity (0.47–0.99) and the RMSE was between 3.57° and 13.14°. The difference between discrete parameters was lower than 14.54°. The repeatability analysis of waveforms showed that the CMC was between 0.54 and 0.95 and the RMSE was less than 5° in the frontal and transverse planes. The ICC of all joint angles in the IMU was general to excellent (0.57–1). Our findings showed that the IMU system might be utilized to evaluate lower extremity 3D joint kinematics in functional motions.

## 1. Introduction

The evaluation of functional tasks has become an essential aspect of sports medicine and physical therapy and has aroused extensive research interest [1]. The assessment of joint kinematics, such as speed, angle and, acceleration during the execution of functional tasks, can not only provide diagnostic information about the recovery status or injury risk but also help with decision making for later rehabilitation, such as evaluation before and after treatment, and comparing different treatment options [2]. Therefore, accurate joint kinematics measurements taken while executing functional tasks may be useful in objectively evaluating actual performance.

In clinical medicine, optoelectronic motion capture is currently considered as the gold standard for human kinematics measurement and quantification [3]. However, the optoelectronic system is expensive, resource-intensive, and largely immobile, and is usually operated in a laboratory with rigorous environmental requirements [4], which limits their clinical application. In the past few decades, inertial sensor technology has emerged as an alternative to three-dimensional motion analysis, and their use has been widely documented [5,6,7]. The inertial measurement unit (IMU) is an electronic device made up of accelerometers, gyroscopes, and magnetometers. These inertial sensors, which are attached to different regions of the body, measure linear acceleration, angular velocity, and the strength of the magnetic field. Using particular sensor fusion algorithms, each inertial sensor unit might provide an estimate of the body segment orientation relative to a global frame of reference. The kinematics of joint movement can be computed when integrated with other sensor units on nearby body segments [8,9]. Compared with an optoelectronic system, these sensor units are small in size, low in cost, and light in weight, and the main advantages are portability and ease of use [10]. With the increasing popularity of wearable sensors, an increasing number of studies have examined their validity and reliability in motion analysis. Meanwhile, these IMU sensors have been successfully used for gait analysis [11,12,13], lower limb joint and pelvic angle kinematics [14,15], upper limb motion analysis [16], and whole body motion analysis [17].

A wide range of commercially available IMU systems have been dedicated to motion analysis, such as Xsens, IMeasureU, BioSyn Systems, and Shimmer Sensing. The accuracy of 3D kinematics has been verified among those systems [18,19]. The Perception Neuron^®^ system (NOITOM, Beijing, China) has been widely applied within virtual reality interaction, visual effects in filmmaking, television development and production, medical diagnosis, and rehabilitation robot control [20,21,22], and has proven to be useful for simulation-based training of surgical trainees [23]. This system estimates 3D joint kinematics using specific biomechanical models and proprietary algorithms. The research results of Sers et al. showed that compared with the VICON motion analysis system, the system deviation of upper limb joint motion range (ROM) calculated by the IMU system was ≤4.5°, the random deviation was −4.5–4.5, and the CMCs were both at 0.99 [24].

Such findings also suggested that the IMU system could be employed to quantify upper limb movements. However, to the best of our knowledge, there has been limited information available about the system’s performance in 3D joint kinematic measurements when evaluating a wider range of motion and more complex motion tasks, such as gait (GA), squat (SQ), single-leg squat (SLS), forward lunge (FL), side lunge (SL), and counter-movement jump (CMJ), which are essential for patients with lower extremity dysfunction, especially osteoarthritis [25]. Secondly, the reliability of the IMU system in measuring human motion has rarely been studied.

Therefore, the primary purpose of this study was to determine the concurrent validity of joint angular kinematics provided by the Perception Neuron^®^ system (NOITOM, Beijing, China) against data obtained using a camera-based optoelectronic system for functional tasks. The secondary objective was to investigate the test–re-test repeatability of the kinematic waveforms and the discrete parameters measured by the IMU system. Our hypothesis was that the IMU measurements of functional motion had good validity and reliability. 

## 2. Materials and Methods

### 2.1. Participants

Twenty healthy participants (10 female, 10 men; age: 25.19 ± 2.8 years; height: 171.85 ± 8.88 cm; body mass: 65.01 ± 12.03 kg) participated in this study. The participants of this study were initially recruited online by filling out questionnaires, and the final participants were determined by the following exclusion criteria: suffering from or previously had neurological, cardiovascular, or musculoskeletal disorders. The study was approved by the Ethics Committee of Beijing Sport University (2020130H) and met the criteria of the Declaration of Helsinki. All participants signed informed consent forms prior to the study.

### 2.2. Instrumentation

*Optoelectronic system*. An OptiTrack system (Prime17W, Natural Point, Corvallis, OR, USA) consisting of eight cameras was used as the gold standard reference system. The calibration was performed according to the OptiTrack user guide instructions [26]. At the beginning of every test, the rater used standardized palpation to place 19 reflective markers (14 mm in diameter) at the anatomical landmarks of the participants according to the Helen Hayes lower limb modeling protocol provided by the software—sacrum, bilateral anterior superior iliac spine, lateral thigh, lateral epicondyle and medial epicondyle of knee joint, lateral calf, lateral malleolus and medial malleolus, and heel and second metatarsal [27]. The reflective markers were fixed in place with medical grade double-sided tape. Prior to the trails, a static calibration test in a neutral standing position was recorded.

*Inertial sensor system.* The Perception Neuron^®^ system consisted of seven wireless IMUs, each (12.5 mm × 13.1 mm × 4.3 mm) and containing a 3-axis gyroscope, 3-axis magnetometer, and 3-axis accelerometer. The system could capture calibrated whole-body inertial motion in real time while streaming and saving kinematic data into its proprietary software. A three-dimensional reconstruction of the human body could be built in the system’s proprietary software, and when calibrated, the wearer’s coherent movement across all body parts could be shown. The IMUs were put on the sacrum, bilaterally on the upper thigh (between the greater trochanter and medial epicondyle of the knee), the lower shank (medial surface of the proximal tibia), and the dorsum of the foot. They were fastened to anatomical landmarks by elastic straps to ensure their position and minimize any movement. Calibration was performed using the manufacturer’s recommended calibration procedure prior to trails: (1) standing in an A pose with shoulders neutral and hands down at the sides of the legs; (2) standing in an S posture with knees flexed by about 45° and shoulders flexed by 90°, with palms facing the floor. According to the calibration standards, each position had to be maintained for a few seconds [20].

The experimental area was cleansed of metal items before the test to guarantee that the IMU’s magnetometer was not impacted by strong magnetic fields, which would reduce the accuracy of the motion capture data.

### 2.3. Experimental Protocol

On two separate days, each participant experienced two sessions. Anthropometric measurements of the participants’ right lower limb in a standing posture as well as height and weight were collected during the first session. At each session, the same rater placed reflective markers at the anatomical landmarks of the participants and helped them wear the sensors.

**Figure 1:**
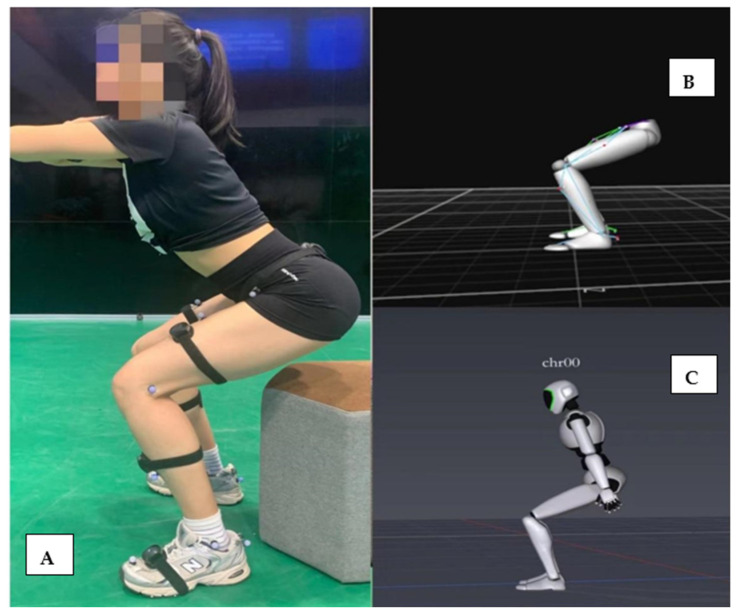
(**A**) Photograph of a participant wearing reflective markers and the Perception Neuron^®^ device during a squat trial. (**B**) A screenshot of the software program that was used to obtain motion data information from the OptiTrack device. (**C**) A screenshot of the software program that was used to obtain motion data information from the Perception Neuron^®^ device.

Before performing each task, the rater demonstrated the motion to the participants and was allowed to ask any questions. After a warm-up, each participant completed the following six tasks in sequence and repeated them three times: gait (GA), squat (SQ), single-leg squat (SLS), side lunge (SL), forward lunge (FL), and counter-movement jump (CMJ). During the SQ and CMJ, to prevent occlusion of the reflecting markers of the anterior superior iliac spine when participants squatted, a chair was positioned behind the individual at a height of about 5 cm above the knee line (see Figure 1 for the example of a participant during a squat). During the FL and SL, the distance between the feet was 70% of the length of the lower leg [1]. For the SLS, FL, and SL tasks, we adopted and imitated the scheme described by Dingenen et al. The participants used the dominant side of the lower limbs, squatted for 2 s, and returned to an upright position for 2 s while maintaining balance [28]. This low movement speed was selected to minimize the chance for trajectory gaps [29]. Participants conducted the GA, SQ, and CMJ tasks at their own pace to best imitate how these activities would be performed in a clinical environment. To lessen the influence of motion speed on joint angles and lower limb kinematics, we used a metronome to provide audio cues for motion speed. The movement cycle of SQ, SLS, FL, and SL was defined as the time from one maximum knee extension to the next; the CMJ cycle was defined as the time from the first downward movement of the pelvic marker to the next static phase; and the gait cycle was defined as the time from one heel hitting the ground to the next (the heel strike time was determined according to the video of the model displayed by the software combined with the data). Every trial was time normalized to 100% of the movement cycle.

### 2.4. Data Processing and Analysis

The reconstruction and auto-labeling of the marker trajectories were originally performed with the Motive2.1 (the unified software platform of OptiTrack) for the data gathered by the OptiTrack system. Each trial was visually examined, and any unmarked tracks were manually noted. Depending on the size and position of the gap, the spline, pattern, and rigid body fill were used to fill it. The data were filtered using a 6 Hz low-pass fourth-order Butterworth filter and were exported as C3D files. The Cortex software (MathWorks Inc., Natick, MA, USA) was used to compute 3D kinematics of the ankle, knee, and hip joints. For the data collected by the IMU system, they were filtered using the Kalman filter by the Axis Studio software (NOITOM, Beijing, China and exported in the format of BVH files. The files were imported into the patent algorithm library of the IMU system, and the data of the gyroscope, magnetometer, and accelerometer were calculated through the fusion algorithm of the system to generate the angles. The positive and negative values of the joint angles calculated by the OptiTrack and IMU systems were not consistent, therefore they were processed for consistency before further analysis (hip flexion, knee flexion, and ankle dorsiflexion were all represented by positive angles). Both the IMU and OptiTrack systems collected the data simultaneously at 100 Hz and manually ensured that they were started at the same time. The knee flexion and extension angles of each experimental data were selected as the basis for the peak detection algorithm. The angular curves obtained by both systems were aligned at the maximum knee flexion angle [18,24] and trimmed to the same time range in Python (version 3.9).

### 2.5. Statistical Analysis

SPSS (version 25.0, IBM Corporation, Armonk, NY, USA) and Python (version 3.9) were used for all statistical analyses. The data were analyzed for each task, each joint (hip, knee, ankle), each plane (sagittal, frontal, transverse), and both sides of the body (left, right). Each gait trial was about 4 to 5 m (about 3 steps). The first and last steps were removed from the analysis to avoid the potential effect of gait initiation (acceleration) and stopping (deceleration) from impacting the analysis, therefore the second step was finally included. The means of the parameters of all tasks were determined and utilized for each participant, system, and joint.

The following parameters were calculated to compare the joint kinematics calculated by the IMU system with the reference system. These consisted of: root mean square error (RSME), coefficient of multiple correlation (CMC), and difference analysis in discrete parameters. The RMSE of waveforms generated by the two systems was regarded as an overall measure of waveform consistency, while the CMC was used to evaluate the waveform similarity [30]. To evaluate the effect of the offset on waveform similarity, CMC was recomputed after zeroing offset. The offset was calculated as the average of the signal over the entire period of motion. The CMC before and after migration was labeled as CMC1 and CMC2, respectively [7]. The value of CMC was rated as poor (≤0.39), fair-to-high (0.40–0.74), good (0.75–0.84), very good (0.85–0.94), or excellent (0.95–1) [7,18]. The difference analysis was used to assess the deviation between systems in discrete parameters, including maximum angle, minimum angle, and range of motion (ROM). To compare the repeatability of the Perception Neuron^®^ system, RMSE and CMC were calculated. Additionally, the intraclass correlation coefficient (ICC) with a two-way random model for consistency was calculated for the discrete parameters in all tasks. ICC ≥ 0.75 indicated excellent repeatability, ICC 0.4–0.74 indicated fair to high repeatability, and ICC ≤0.39 indicated poor repeatability [31].

## 3. Results

Twenty participants completed the two-day tests. The kinematics of a total of 720 functional trials (20 participants × 6 tasks × 3 times × 2 systems) in each session were analyzed. As the data showed similar results on both sides of the GA, SQ, and CMJ, the dominant side of the participants was selected to display the results.

### 3.1. Concurrent Validity

**Figure 2:**
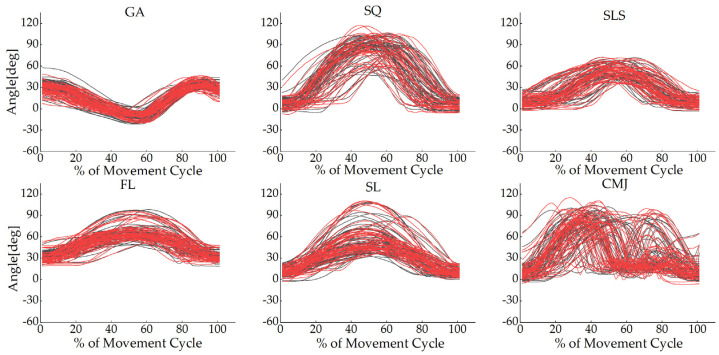
Hip joint angles in the sagittal plane for each participant in all tasks during the movement cycle of the gait (GA), squat (SQ), single-leg squat (SLS), side lunge (SL), forward lunge (FL), and counter-movement jump (CMJ). The black lines represent the OptiTrack system, while the red lines represent the Perception Neuron^®^ system. The *Y*-axis depicts joint angles in degrees, while the *X*-axis depicts the movement cycle in %.

**Figure 3:**
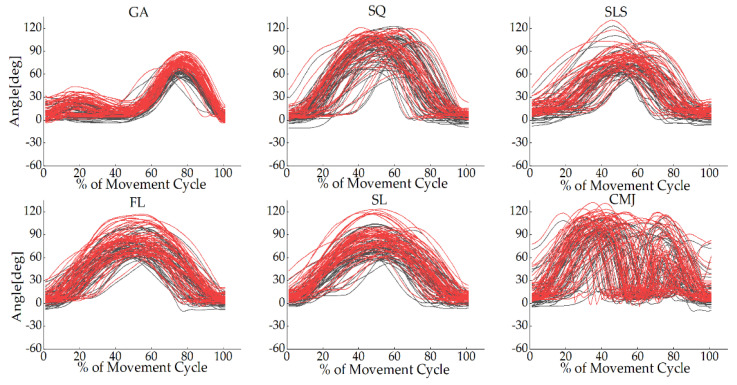
Knee joint angles in the sagittal plane for each participant in all tasks during the movement cycle of the gait (GA), squat (SQ), single-leg squat (SLS), side lunge (SL), forward lunge (FL), and counter-movement jump (CMJ). The black lines represent the OptiTrack system, while the red lines represent the Perception Neuron^®^ system. The *Y*-axis depicts joint angles in degrees, while the *X*-axis depicts the movement cycle in %.

**Figure 4:**
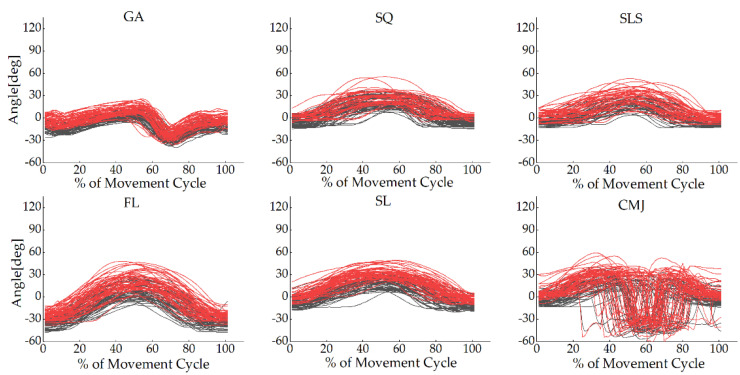
Ankle joint angles in the sagittal plane for each participant in all tasks during the movement cycle of the gait (GA), squat (SQ), single-leg squat (SLS), side lunge (SL), forward lunge (FL), and counter-movement jump (CMJ). The black lines represent the OptiTrack system, while the red lines represent the Perception Neuron^®^ system. The *Y*-axis depicts joint angles in degrees, while the *X*-axis depicts the movement cycle in %.

**Figure A1:**
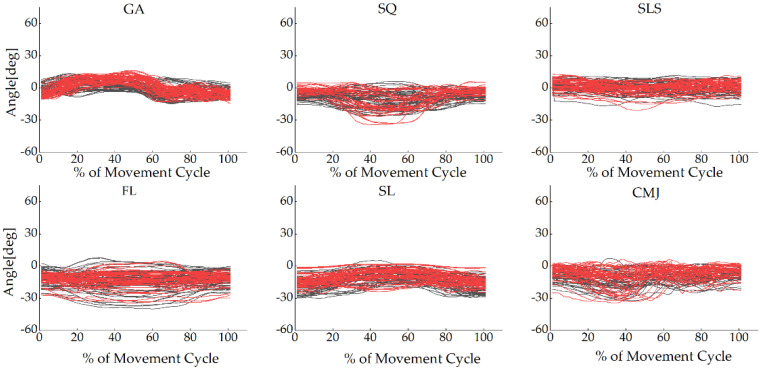
Hip joint angles in the frontal plane for each participant in all tasks during the movement cycle of the gait (GA), squat (SQ), single-leg squat (SLS), side lunge (SL), forward lunge (FL), and counter-movement jump (CMJ). The black lines represent the OptiTrack system, while the red lines represent the Perception Neuron^®^ system. The *Y*-axis depicts joint angles in degrees, while the *X*-axis depicts the movement cycle in %.

**Figure A2:**
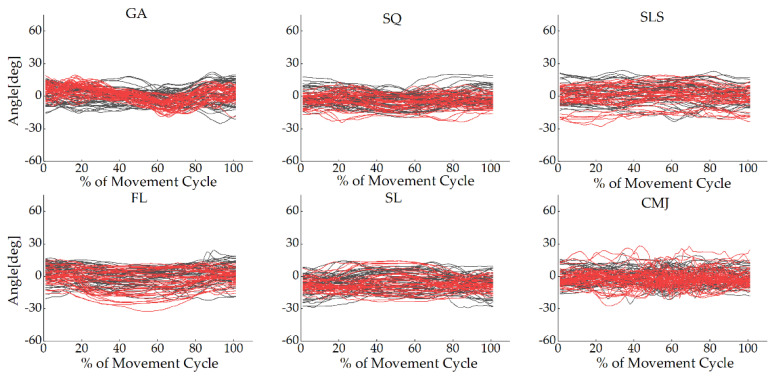
Hip joint angles in the transverse plane for each participant in all tasks during the movement cycle of the gait (GA), squat (SQ), single-leg squat (SLS), side lunge (SL), forward lunge (FL), and counter-movement jump (CMJ). The black lines represent the OptiTrack system, while the red lines represent the Perception Neuron^®^ system. The *Y*-axis depicts joint angles in degrees, while the *X*-axis depicts the movement cycle in %.

**Figure A3:**
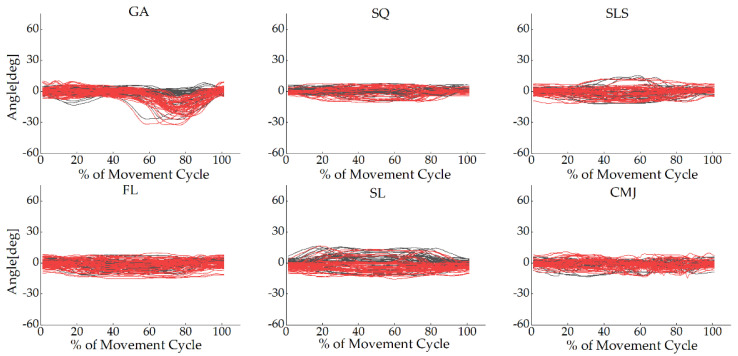
Knee joint angles in the frontal plane for each participant in all tasks during the movement cycle of the gait (GA), squat (SQ), single-leg squat (SLS), side lunge (SL), forward lunge (FL), and counter-movement jump (CMJ). The black lines represent the OptiTrack system, while the red lines represent the Perception Neuron^®^ system. The *Y*-axis depicts joint angles in degrees, while the *X*-axis depicts the movement cycle in %.

**Figure A4:**
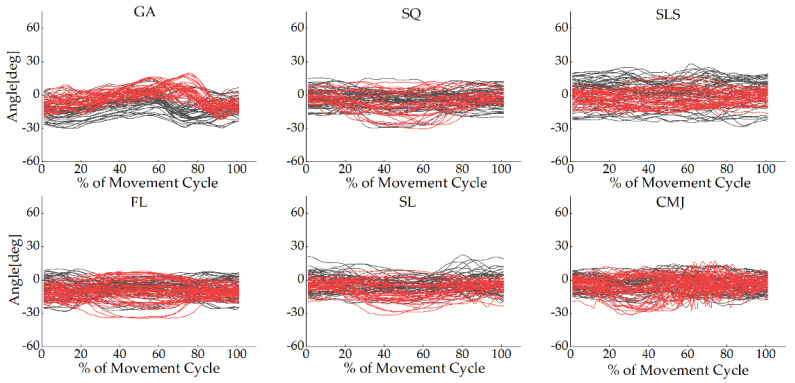
Knee joint angles in the transverse plane for each participant in all tasks during the movement cycle of the gait (GA), squat (SQ), single-leg squat (SLS), side lunge (SL), forward lunge (FL), and counter-movement jump (CMJ). The black lines represent the OptiTrack system, while the red lines represent the Perception Neuron^®^ system. The *Y*-axis depicts joint angles in degrees, while the *X*-axis depicts the movement cycle in %.

**Figure A5:**
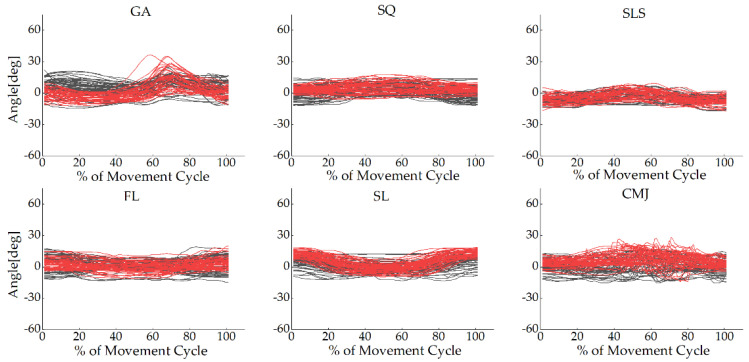
Ankle joint angles in the frontal plane for each participant in all tasks during the movement cycle of the gait (GA), squat (SQ), single-leg squat (SLS), side lunge (SL), forward lunge (FL), and counter-movement jump (CMJ). The black lines represent the OptiTrack system, while the red lines represent the Perception Neuron^®^ system. The *Y*-axis depicts joint angles in degrees, while the *X*-axis depicts the movement cycle in %.

**Figure A6:**
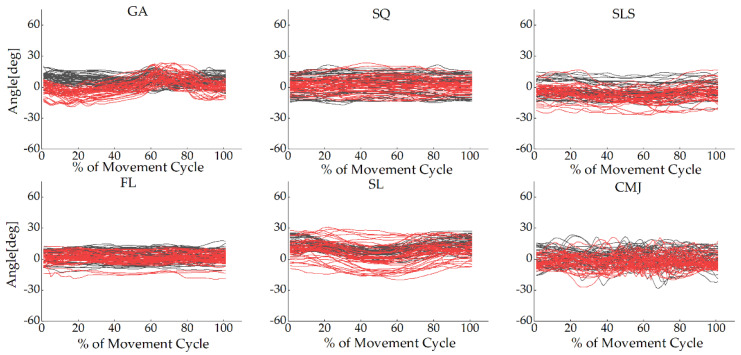
Ankle joint angles in the transverse plane for each participant in all tasks during the movement cycle of the gait (GA), squat (SQ), single-leg squat (SLS), side lunge (SL), forward lunge (FL), and counter-movement jump (CMJ). The black lines represent the OptiTrack system, while the red lines represent the Perception Neuron^®^ system. The *Y*-axis depicts joint angles in degrees, while the *X*-axis depicts the movement cycle in %.

The joint angle waveforms for all tasks are shown in Figure 2, Figure 3 and Figure 4 and Appendix A, Figure A1, Figure A2, Figure A3, Figure A4, Figure A5 and Figure A6. The angles measured from the two systems in the sagittal plane were similar to each other, but the other planes were different. The absolute value offset provided by these two systems was clear, particularly for the knee and ankle in the sagittal plane. This observation was consistent across all tasks.

#### 3.1.1. Waveform Analysis

**Figure 5:**
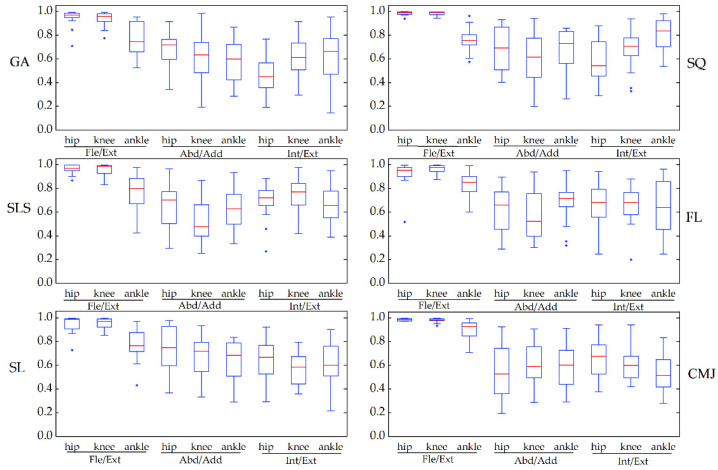
The waveform similarity between Perception Neuron^®^ system and OptiTrack system data before offset removal (CMC1) was assessed using box-and-whiskers plots of CMC values in the gait (GA), squat (SQ), single-leg squat (SLS), side lunge (SL), forward lunge (FL), and counter-movement jump (CMJ). Fle/Ext stands for “Flexion/Extension”, Abd/Add for “Abduction/Adduction”, and Int/Ext for “Internal/External Rotation”.

**Figure 6:**
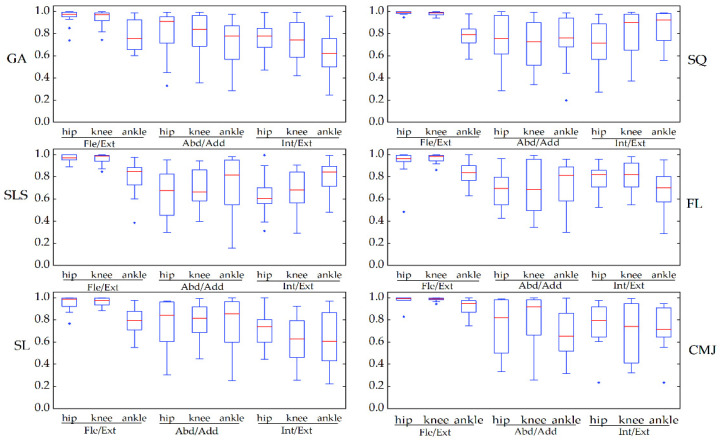
The waveform similarity between Perception Neuron^®^ system and OptiTrack system data after offset removal (CMC2) was assessed using box-and-whiskers plots of CMC values in the gait (GA), squat (SQ), single-leg squat (SLS), side lunge (SL), forward lunge (FL), and counter-movement jump (CMJ). Fle/Ext stands for “Flexion/Extension”, Abd/Add for “Abduction/Adduction”, and Int/Ext for “Internal/External Rotation”.

Figure 5 depicts the similarity of joint angle waveforms for the two systems for each task. The coefficient of multiple correlation before offset (CMC1) of all the tasks in the sagittal plane ranged from 0.76 to 0.99, good to excellent, and among this, the CMC1 of the hip and knee joints was greater than 0.92. The CMC1 of the joint angle waveforms in the frontal and transverse planes was lower, ranging from 0.52 to 0.73 and from 0.47 to 0.81, respectively. From the box plots, the CMC1 showed the same similarity as the observed joint angle waveforms in all tasks. The coefficient of multiple correlation after removing the offset (CMC2) between the two systems’ motion waveforms resulted in an increase, ranging from 0.78 to 0.99 in the sagittal plane, from 0.65 to 0.87 in the frontal plane, and from 0.62 to 0.81 in the transverse plane (see Figure 6).

The root mean square error (RMSE) of the two systems ranged from 3.57° to 13.14° for all the joints. The highest RMSE was shown in the sagittal plane of the ankle between 8.79° and 13.14°. Overall, the RMSE of the frontal plane of the three joints, ranging from 3.57° to 9.08°, was smaller than that of the sagittal and transverse planes, ranging from 5.20° to 13.14° and from 5.98° to 10.80°, respectively. After offset correction, the RMSE of all the joints ranged from 2.08° to 12.83° (see Table 1).

#### 3.1.2. Difference Analysis

**Figure 7:**
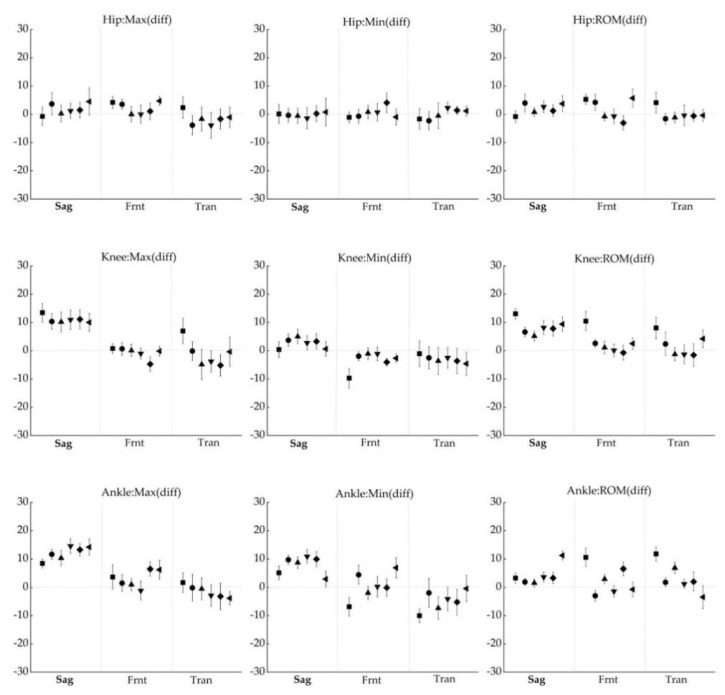
Difference between IMU suit and OptiTrack system in minimum angle (left row), maximum angle (center row), range of motion (right row). The six data points of each plane correspond to the GA (■), SQ (●), SLS (▲), FL (▼), SL (◆), and CMJ (◀). Positive numbers showed that the angle calculated by the Perception Neuron^®^ system was larger than the angle computed by the OptiTrack system. The error bars represent the 95% confidence interval of the difference.

The differences in joint angles provided by the IMU system and the OptiTrack system at discrete parameters are shown in Figure 7. In all tasks, the deviation of maximum, minimum, and ROM in the three planes was lower than 5.72° for the hip, 13.45° for the knee, and 14.54° for the ankle.

### 3.2. Reliability

**Figure 8:**
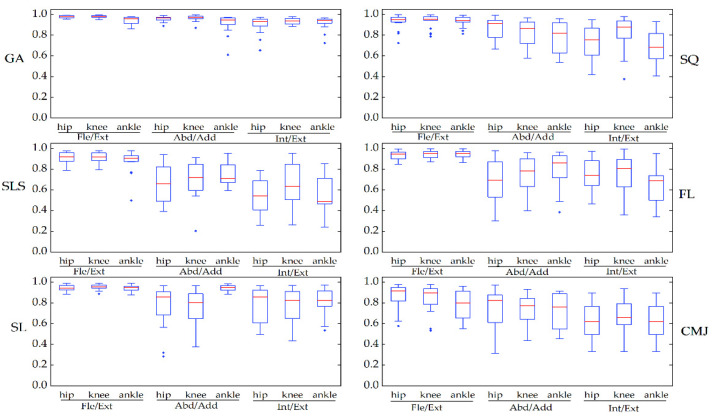
The waveform similarity in the Perception Neuron^®^ system between two days was examined using box-and-whiskers plots of CMC values in the gait (GA), squat (SQ), single-leg squat (SLS), side lunge (SL), forward lunge (FL), and counter-movement jump (CMJ). Fle/Ext stands for “Flexion/Extension”, Abd/Add for “Abduction/Adduction”, and Int/Ext for “Internal/External Rotation”.

**Figure A7:**
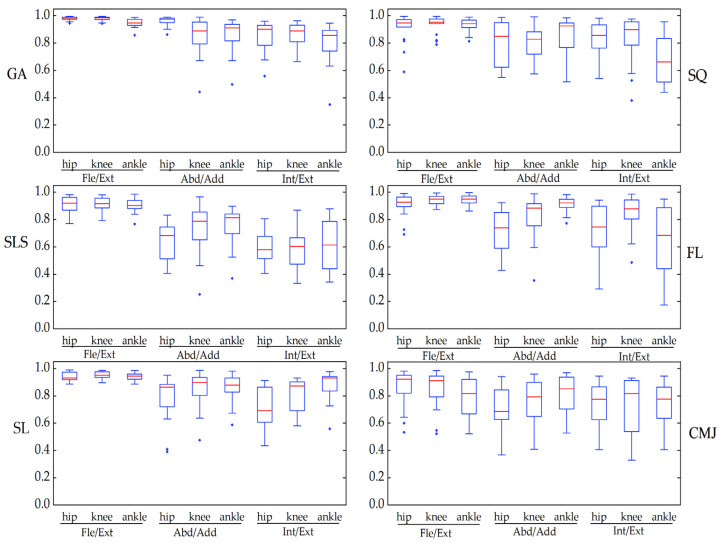
The waveform similarity in the OptiTrack system between two days was examined using box-and-whiskers plots of CMC values in the gait (GA), squat (SQ), single-leg squat (SLS), side lunge (SL), forward lunge (FL), and counter-movement jump (CMJ). Fle/Ext stands for “Flexion/Extension”, Abd/Add for “Abduction/Adduction”, and Int/Ext for “Internal/External Rotation”.

The CMC of the kinematic waveforms for all angles in GA ranged from very good to excellent (between 0.91 and 0.98). In the other tasks, the CMC ranged from 0.79 to 0.95 in the sagittal plane, from 0.66 to 0.94 in the frontal plane, and from 0.54 to 0.83 in the transverse plane (see Figure 8). The RMSE of the waveforms was between 4.14° and 21.34° in the sagittal plane, was the highest (15.18°–21.34°) among the CMJ, and was less than 3.45° in the frontal plane and 4.61° in the transverse plane. The changes in CMC and RMSE in the optical system were similar to those in the IMU system (Table 2 and Appendix B, Figure A7).

The intraclass correlation coefficient (ICC) of all joint angles in the IMU was fair to excellent (ICC between 0.57 and 1) (see Table 3). These results were basically consistent with the comparisons against the optical system (ICC between 0.64 and 0.99), as presented in Appendix B Table A1.

## 4. Discussion

The primary goal of this study was to assess the accuracy of the Perception Neuron^®^ system in measuring lower limb kinematics using the optical system as a gold reference. Our results showed that there was a strong correlation between the two systems in assessing the joint angles in the sagittal plane and an acceptable correlation between the joint angles in the frontal and transverse planes. The second goal of the study was to assess the re-test reliability of the kinematic waveform and the discrete parameters recorded by the Perception Neuron^®^ system. The results revealed fair to excellent correlations of waveforms between the testing and re-testing of the IMU system, and the discrete parameters (such as maximum, minimum, and ROM) were general to excellent.

### 4.1. Concurrent Validity

In all the tasks, our results showed high CMC values in the sagittal plane, indicating that the IMU system and the optical system had highly similar waveforms in this plane. The CMC of the waveforms in the frontal and transverse planes revealed that the two systems evaluated adduction/abduction and internal/external rotation angles with fair to excellent correlation. Some studies have compared the joint angles obtained from the optical system of a typical model using anatomical landmarks and the joint angles obtained from IMUs [11,18]. Zhang et al. [11] reported that the CMC was 0.96 or higher for the flexion/extension waveforms and from 0.5 to 0.85 in the other two rotational axes during walking trials on the ankle, knee, and hip angles. Al-Amri et al. [18], in a validity study of clinical practice, used R^2^ as an alternative measure of similarity for CMC and observed R^2^ > 0.8 for sagittal plane angles, with this ranging from 0.4 to 0.8 for transverse and frontal plane angles during walk, squat, and jump. Our results yielded CMC values similar to those studies, and our results extend these previous findings by including richer dynamic tasks.

Although the waveform of the joint angles had good to excellent similarity in the sagittal plane, the RSME was higher. In our study, the RMSE was between 5.20° and 13.14° in the sagittal plane and between 2.95° and 10.97° in the frontal and transverse planes. Our observed RMSE was similar to Takeda et al. [32] in that the RSME ranged from 6.45° to 10.34° in hip and knee flexion/extension and from 4.10° to 5.55° in hip abduction/adduction. This result was also better than that of van den Noort; they compared the gait of children with cerebral palsy between the two systems, with an RMSE between 4.6° and 16.1° [33]. Cloete et al. [34] compared lower limb joint angles during gait that was evaluated simultaneously by the IMU and optical systems and reported the RMSE of the joint angle as even up to 27.4° without offset correction and from 5.71° to 18.88° with offset correction in all joint angles. However, in other studies, when the optical kinematics data calculated using the marker clusters are compared with the inertial sensor, the RMSE was smaller than our own [35,36]. Teufl et al. calculated the joint angles when the reflective markers were placed in a marker cluster and the anatomic positions were compared with the angles calculated by the IMU system. The RMSE and range of motion error were significantly greater when the markers were placed at anatomic positions than when they were placed on marker clusters, with an average increase of around 2° to 3° for all tasks [37]. This difference might be partly due to the placement of the reflective markers. 

The optical system and the IMU system, as different collection and analysis tools of kinematic data, have many differences. The differences between the two observed in this study were particularly related to their biomechanical model. Different biomechanical models had different definitions of the anatomical framework. The primary effect of anatomical framework differences on the sagittal plane joint angle was offset, while the waveform was similar, and the waveform distortion of the other two axes had a more complex relationship [38,39]. For example, the optical system constructed segmental frames to calculate angles based on anatomical locations by markers, while the IMU system determined the frames based on directions associated with calibrated positions. Especially when the joint angles in the frontal and transverse planes were moving within a small range, the effect of the anatomical framework might be more obvious. The high RSME in this study was also related to model differences. We compared the RSME calculated by the same model (using the IMU and marker clusters to calculate the kinematics from the segment position data) and different models (using the IMU and anatomical models to calculate the kinematics) and found that the RSME calculated by the same model was smaller. This result not only emphasizes the importance of location or motion data sources (inertial sensors and cameras), but also the importance of the model used for measurement. Therefore, many confirmatory studies of inertial sensors placed the markers directly on the IMU rather than on soft tissue anatomical points in order to reduce measurement errors [37,40]. These studies, however, only examined the measurement accuracy of optical and inertial systems. To acquire an accurate representation of the IMU system’s performance, we decided to put reflective markers on anatomical markers to test the IMU’s capacity to monitor specific human motions rather than the sensor’s absolute precision. In addition to the influence caused by the biomechanical model, the optical system and the IMU system themselves had measurement errors. Optical systems based on placing reflective spots on the skin might be more susceptible to soft tissue artifact (STA) [41]. Skin deformations caused the markers to be displaced relative to the underlying bone while employing optoelectronic stereophotogrammetry. The movement of skin markers relative to their underlying bone (STA) undermined the accuracy of marker-based motion analysis [42,43]. In addition, mismatches between the position of actual markers and the modeled position might lead to errors in the calculation of joint angles [44]. For the IMU system, the measured kinematics might contain sensor-to-segment calibration errors due to a mismatch between the practiced N or T pose and the modeled pose. Moreover, the possible defects in model scaling might be another source of error that affects accuracy. Table 4 summarizes the advantages and disadvantages of the IMU and optical systems.

In order to characterize the motion pattern, it was usually necessary to calculate discrete parameters such as the maximum and minimum angles of joint movement as well as its ROM. Our results showed that the angle difference of the knee and ankle joints in the sagittal plane was larger. The IMU system systematically overstated the sagittal plane joint angles of the knee and ankle. There were offsets between the systems, as described in the waveform, explaining some of the differences between the minimum and maximum joint angles. This meant that the discrete parameters of the IMU system and the optoelectronic system could not be directly compared. Furthermore, we discovered systematic changes in ROM that were connected to offset discrepancies across the systems.

### 4.2. Reliability

When comparing data from the test and re-test, the CMC for all gait joint angles measured by the IMU system ranged from very good to excellent, which was equivalent to the findings of a systematic review summarizing the reliability of photoelectric 3D gait analysis [45]. Our study complemented the reliability results of other tasks in that the CMC ranged from very good to excellent in the sagittal plane and fair to very good in the frontal and transverse planes. In all tasks, the RMSE of the waveform was smaller than 5° in the frontal and transverse planes, and was relatively high in the sagittal plane. Especially in the CMJ, the RMSE was greater than 15°. This might be due to the wide range of motion of the joint angle in the sagittal plane, which resulted in a considerable variation in angles between the testing of two days. The optoelectronic system provided similar results suggesting that the variability of the motion itself was larger. Several previous studies using inertial sensors to measure joint angles had shown that the intraclass correlation coefficient (ICC) of re-test measurements was good [18,41], and that lower extremity joint angles in the sagittal plane had more consistent reliability than in the frontal or transverse planes [13,46]. Our results showed that both the ICC of lower extremity joint angles and the ROM were excellent in the reliability evaluation, with exception of the ROM of the hip and ankle for the single-leg squat in the transverse plane, which was general to high, and such a phenomenon might also be attributed to the variability of the motion caused by the instability of the movement itself.

Overall, the repeatability of the IMU system was comparable to that of the optoelectronic system, demonstrating clinically acceptable repeatability. Due to the broad range of motion of the hip, knee, and ankle joints in the sagittal plane of the functional tasks, as well as the motor variability caused by instability, the relevant measurements should be interpreted with caution.

### 4.3. Limitations

Although the squat height was restricted during the SQ and CMJ to avoid occluding the reflecting markers of the anterior superior iliac spine, it was not guaranteed that the participants would have the same range of motion in the sagittal plane on two different days, which might be the reason for the large root mean square error of the SQ and CMJ in the re-test measurements. Furthermore, due to the close proximity between the marker placed in the center of the posterior superior iliac spine and the IMU placed in the sacrum, they might be mutually squeezed during large movement of ROM (e.g., SQ and CMJ), thus causing their positions to be slightly shifted. Finally, all the participants in this study were healthy adults, and further studies are needed to validate the system with pathological populations or elite athletes.

## 5. Conclusions

In summary, this paper examined the Perception Neuron^®^ system to assess its validity and reliability as a potential replacement for the optoelectronic system, with low costs, simpler procedures, and few space constraints. The results showed that an IMU-based motion analysis system could be effectively applied to clinical functional movement analysis. As a result, the IMU-based system provided accurate functional action data that could help doctors, sports scientists, and fitness trainers diagnose and evaluate more quickly.

## Figures and Tables

**Table 1:** Mean root mean square error of Perception Neuron^®^ and OptiTrack systems in all tasks.

		Hip			Knee			Ankle		
		Fle/Ext	Abd/Add	Int/Ext	Fle/Ext	Abd/Add	Int/Ext	Fle/Ext	Abd/Add	Int/Ext
RSME (before)	GA	6.48 ± 3.51	6.26 ± 2.48	8.91 ± 2.44	9.29 ± 4.59	5.97 ± 3.05	10.80 ± 4.57	8.79 ± 3.25	9.08 ± 3.60	10.18 ± 3.11
	SQ	6.32 ± 2.39	4.66 ± 1.88	7.08 ± 4.46	11.04 ± 7.10	4.09 ± 2.38	7.29 ± 3.42	10.93 ± 3.12	6.27 ± 3.99	8.77 ± 6.10
	SLS	5.20 ± 3.02	5.62 ± 3.43	8.42 ± 4.55	9.12 ± 6.32	4.09 ± 1.76	10.97 ± 3.74	9.78 ± 4.88	4.40 ± 2.09	8.40 ± 5.52
	FL	5.85 ± 3.02	5.59 ± 3.34	9.31 ± 5.64	9.49 ± 5.50	4.22 ± 2.33	7.49 ± 4.72	13.14 ± 4.51	5.99 ± 3.77	5.98 ± 3.02
	SL	5.36 ± 2.77	6.10 ± 3.94	8.21 ± 3.23	10.36 ± 6.05	5.66 ± 2.72	9.00 ± 4.94	11.64 ± 3.56	6.90 ± 2.96	9.48 ± 5.33
	CMJ	7.44 ± 5.82	5.55 ± 1.85	8.31 ± 4.19	9.16 ± 4.59	3.57 ± 1.56	9.75 ± 5.60	12.12 ± 4.44	8.28 ± 4.30	8.25 ± 2.98
RSME (after)	GA	5.49 ± 4.07	3.29 ± 2.40	5.15 ± 3.34	7.83 ± 5.05	3.56 ± 3.20	7.54 ± 5.82	8.31 ± 3.52	6.87 ± 4.48	7.09 ± 3.98
	SQ	4.96 ± 2.43	2.95 ± 2.25	5.42 ± 4.89	8.02 ± 5.13	3.03 ± 2.47	4.97 ± 4.37	10.78 ± 3.23	5.40 ± 4.47	8.16 ± 6.50
	SLS	4.69 ± 3.31	4.54 ± 3.44	7.20 ± 5.03	8.42 ± 6.34	3.06 ± 1.83	9.95 ± 4.26	9.68 ± 4.86	3.40 ± 2.45	6.59 ± 6.40
	FL	5.17 ± 3.34	4.69 ± 3.35	7.46 ± 6.22	8.19 ± 5.79	3.34 ± 2.55	6.12 ± 5.12	12.83 ± 4.67	5.28 ± 4.09	5.24 ± 3.38
	SL	4.52 ± 3.24	5.10 ± 4.22	6.21 ± 4.16	9.29 ± 6.20	4.74 ± 2.97	7.40 ± 5.20	11.38 ± 3.68	5.46 ± 3.41	8.33 ± 5.74
	CMJ	5.59 ± 5.32	3.58 ± 2.35	6.03 ± 4.68	7.77 ± 4.90	2.08 ± 1.92	7.13 ± 6.59	11.07 ± 4.86	6.34 ± 4.70	5.53 ± 3.65

**Table 2:** The RMSE of IMU and OptiTrack systems collected in all tasks.

		Hip			Knee			Ankle		
		Fle/Ext	Abd/Add	Int/Ext	Fle/Ext	Abd/Add	Int/Ext	Fle/Ext	Abd/Add	Int/Ext
RSME (IMU)	GA	4.21 ± 1.19	2.32 ± 0.78	2.88 ± 0.89	6.33 ± 2.19	1.82 ± 0.69	3.29 ± 0.85	4.14 ± 1.24	2.91 ± 1.04	3.00 ± 0.61
	SQ	11.78 ± 3.72	2.28 ± 1.01	2.39 ± 0.69	13.87 ± 4.92	1.59 ± 0.45	2.74 ± 0.97	4.60 ± 1.62	1.48 ± 0.67	1.98 ± 0.49
	SLS	8.92 ± 2.64	2.35 ± 0.83	3.36 ± 1.03	13.81 ± 4.18	1.86 ± 0.64	2.91 ± 0.75	6.56 ± 2.69	3.08 ± 0.95	4.61 ± 1.33
	FL	5.55 ± 1.77	1.93 ± 0.62	3.09 ± 1.53	11.38 ± 4.24	1.59 ± 0.64	2.51 ± 0.73	7.17 ± 2.86	1.77 ± 0.64	2.37 ± 0.77
	SL	6.86 ± 3.15	2.34 ± 0.83	2.82 ± 1.02	10.38 ± 3.45	1.63 ± 0.88	2.61 ± 0.80	5.12 ± 1.65	2.24 ± 0.86	2.96 ± 0.85
	CMJ	15.18 ± 5.63	3.43 ± 1.63	4.00 ± 1.29	21.34 ± 7.60	2.26 ± 0.68	4.28 ± 1.15	16.11 ± 5.82	3.45 ± 1.01	3.96 ± 1.30
RSME (OptiTrack)	GA	4.08 ± 1.48	1.55 ± 0.63	3.08 ± 0.75	5.04 ± 1.99	1.25 ± 0.59	2.95 ± 1.08	3.70 ± 1.12	1.87 ± 0.62	2.23 ± 0.56
	SQ	12.12 ± 5.45	1.97 ± 0.74	2.38 ± 0.92	13.87 ± 6.24	1.13 ± 0.57	2.46 ± 0.85	4.43 ± 1.54	1.66 ± 0.48	1.49 ± 0.52
	SLS	8.59 ± 2.45	3.19 ± 1.62	4.21 ± 1.37	12.50 ± 3.86	1.62 ± 0.82	4.46 ± 1.44	5.37 ± 1.86	2.64 ± 1.57	1.96 ± 0.73
	FL	5.79 ± 2.44	2.64 ± 1.08	3.06 ± 1.58	10.29 ± 3.37	1.26 ± 0.69	2.68 ± 1.14	6.71 ± 2.46	1.69 ± 0.73	1.71 ± 0.55
	SL	6.96 ± 4.04	3.17 ± 1.64	3.33 ± 0.83	9.50 ± 3.38	1.59 ± 0.83	3.22 ± 1.15	4.84 ± 1.46	1.70 ± 0.84	2.11 ± 0.89
	CMJ	15.67 ± 7.19	2.82 ± 0.99	4.21 ± 1.66	19.19 ± 7.06	1.53 ± 0.88	3.70 ± 1.12	13.30 ± 5.44	3.67 ± 1.75	4.55 ± 1.74

**Table 3:** The intraclass correlation coefficient (ICC) of IMU system collected in all tasks.

			GA	SQ	SLS	FL	SL	CMJ
Max	Hip	Fle/Ext	0.97	0.99	0.97	0.99	0.99	0.97
		Abd/Add	0.93	0.98	0.97	0.97	0.98	0.97
		Int/Ext	0.93	0.98	0.98	0.97	0.99	0.97
	Knee	Fle/Ext	0.97	1.00	0.97	0.99	0.99	0.97
		Abd/Add	0.98	0.99	0.99	0.99	1.00	0.99
		Int/Ext	0.98	0.99	0.98	0.98	0.98	0.96
	Ankle	Fle/Ext	0.96	0.99	0.93	0.99	0.97	0.97
		Abd/Add	0.96	0.99	0.94	0.98	0.98	0.94
		Int/Ext	0.94	0.99	0.93	0.98	0.99	0.96
Min	Hip	Fle/Ext	0.98	0.94	0.93	0.97	0.89	0.87
		Abd/Add	0.89	1.00	0.97	0.99	0.97	0.99
		Int/Ext	0.96	0.99	0.98	0.98	0.98	0.95
	Knee	Fle/Ext	0.95	0.93	0.89	0.84	0.92	0.87
		Abd/Add	0.97	0.99	0.98	0.98	0.98	0.97
		Int/Ext	0.94	1.00	0.99	0.99	0.99	0.99
	Ankle	Fle/Ext	0.92	0.98	0.71	0.99	0.95	0.97
		Abd/Add	0.97	0.98	0.91	0.98	0.98	0.97
		Int/Ext	0.91	0.99	0.88	0.99	0.98	0.93
ROM	Hip	Fle/Ext	0.97	0.99	0.93	0.98	0.99	0.94
		Abd/Add	0.94	0.99	0.94	0.95	0.94	0.97
		Int/Ext	0.93	0.94	0.63	0.94	0.92	0.89
	Knee	Fle/Ext	0.92	0.99	0.93	0.98	0.97	0.97
		Abd/Add	0.97	0.94	0.97	0.91	0.99	0.73
		Int/Ext	0.92	0.99	0.89	0.97	0.97	0.96
	Ankle	Fle/Ext	0.91	0.99	0.97	0.99	0.94	0.98
		Abd/Add	0.94	0.96	0.86	0.96	0.98	0.94
		Int/Ext	0.90	0.82	0.57	0.78	0.98	0.90

**Table 4:** Comparison of considered tracking technologies.

Technologies	Pros	Cons
IMU	Simple to use	No precise absolute positioning
	Very lightweight	Extremely magnetically sensitive
	Extremely large recording volume	Calibration errors
	Easy and quick calibration	Positional and rotational errors
	Works just fine over WiFi	
	Almost no disconnecting or data loss during hours of operation	
	Cheaper	
OptiTrack	High precision	Recording volume limited
	Capture rates are high	Markers can be occluded
	Easily recreates complex movement	Extensive post-processing may be necessary to handle marker swap, missing data, and noisy data
		High cost of the hardware

## Data Availability

The accession numbers have not yet been obtained; they will be provided during review.

## Appendix B

**Table A1:** The intraclass correlation coefficient (ICC) of OptiTrack system.

			GA	SQ	SLS	FL	SL	CMJ
Max	Hip	Fle/Ext	0.82	1.00	0.97	0.90	0.99	0.85
		Abd/Add	0.96	0.99	0.92	0.96	0.82	0.99
		Int/Ext	0.92	0.97	0.98	0.98	0.98	0.94
	Knee	Fle/Ext	0.95	0.99	0.98	0.99	0.97	0.96
		Abd/Add	0.98	0.99	0.98	1.00	1.00	0.96
		Int/Ext	0.98	0.99	0.98	0.99	0.98	0.99
	Ankle	Fle/Ext	0.95	0.96	0.97	0.99	0.94	0.98
		Abd/Add	0.93	0.99	0.94	0.99	0.99	0.97
		Int/Ext	0.93	1.00	0.98	0.98	0.98	0.93
Min	Hip	Fle/Ext	0.99	0.91	0.93	0.95	0.93	0.64
		Abd/Add	0.98	0.99	0.91	0.97	0.98	0.97
		Int/Ext	0.89	0.99	0.99	0.98	0.97	0.97
	Knee	Fle/Ext	0.95	0.94	0.95	0.98	0.95	0.92
		Abd/Add	0.99	1.00	0.99	1.00	1.00	1.00
		Int/Ext	0.98	0.99	0.98	0.99	0.97	0.98
	Ankle	Fle/Ext	0.88	0.97	0.98	0.99	0.98	0.93
		Abd/Add	0.98	1.00	0.99	1.00	0.98	0.99
		Int/Ext	0.96	1.00	0.99	0.99	0.98	0.92
ROM	Hip	Fle/Ext	0.78	0.98	0.94	0.93	0.98	0.94
		Abd/Add	0.92	0.98	0.77	0.84	0.84	0.88
		Int/Ext	0.65	0.94	0.78	0.96	0.80	0.91
	Knee	Fle/Ext	0.92	0.98	0.95	0.99	0.96	0.95
		Abd/Add	0.99	1.00	0.95	1.00	0.99	0.99
		Int/Ext	0.87	0.99	0.70	0.99	0.96	0.97
	Ankle	Fle/Ext	0.76	0.95	0.95	0.98	0.91	0.97
		Abd/Add	0.94	0.99	0.89	0.97	0.95	0.95
		Int/Ext	0.67	9.93	0.64	0.90	0.98	0.86
